# Central Administration of Insulin and Leptin Together Enhance Renal Sympathetic Nerve Activity and Fos Production in the Arcuate Nucleus

**DOI:** 10.3389/fphys.2016.00672

**Published:** 2017-01-09

**Authors:** Hamza Habeeballah, Naif Alsuhaymi, Martin J. Stebbing, Trisha A. Jenkins, Emilio Badoer

**Affiliations:** School of Health and Biomedical Sciences, RMIT University, Melbourne, VIC, Australia

**Keywords:** insulin, leptin, renal sympathetic nerve activity, central interactions, cardiovascular function

## Abstract

There is considerable interest in the central actions of insulin and leptin. Both induce sympatho-excitation. This study (i) investigated whether centrally administered leptin and insulin together elicits greater increases in renal sympathetic nerve activity (RSNA), mean arterial pressure (MAP) and heart rate (HR) than when given alone, and (ii) quantified the number of activated neurons in brain regions influencing SNA, to identify potential central sites of interaction. In anesthetised (urethane 1.4–1.6 g/kg iv) male Sprague-Dawley rats, RSNA, MAP, and HR were recorded following intracerebroventricular (ICV) saline (control; *n* = 5), leptin (7 μg; *n* = 5), insulin (500 mU; *n* = 4) and the combination of leptin and insulin; (*n* = 4). Following leptin or insulin alone, RSNA was significantly increased (74 and 62% respectively). MAP responses were not significantly different between the groups. Insulin alone significantly increased HR. Leptin alone also increased HR but it was significantly less than following insulin alone (*P* < 0.005). When leptin and insulin were combined, the RSNA increase (124%) was significantly greater than the response to either alone. There were no differences between the groups in MAP responses, however, the increase in HR induced by insulin was attenuated by leptin. Of the brain regions examined, only in the arcuate nucleus did leptin and insulin together increase the number of Fos-positive cell nuclei significantly more than leptin or insulin alone. In the lamina terminalis and rostroventrolateral medulla, leptin and insulin together increased Fos, but the effect was not greater than leptin alone. The results suggest that when central leptin and insulin levels are elevated, the sympatho-excitatory response in RSNA will be greater. The arcuate nucleus may be a common site of cardiovascular integration.

## Introduction

Insulin is produced by the pancreas and released into the circulation in response to elevated glucose. In addition to insulin's critical role in glucose homeostasis, there is now considerable interest in its role in regulating the autonomic nervous system. Insulin can induce increases in sympathetic outflow to cardiovascular organs, such as the kidney and skeletal muscle vasculature, by activating receptors within the central nervous system (Morgan et al., 2008; Morgan and Rahmouni, 2010).

Leptin is released from adipocytes and it acts centrally to influence metabolic and cardiovascular functions (Elmquist et al., 1997; Mark et al., 1999, 2002; Rahmouni et al., 2005). It acts within the brain to increase sympathetic nerve activity to the kidney (Dunbar et al., 1997; Rahmouni and Haynes, 2004; Rahmouni et al., 2004). The sympatho-excitatory actions of leptin to the kidney makes an important contribution to the abnormally elevated RSNA observed in obesity or overweight conditions (Haynes et al., 1997; Prior et al., 2010).

Both leptin and insulin can be transported across the blood brain barrier to activate brain nuclei involved in cardiovascular and metabolic regulation. Pathways involving the activation of melanocortin 4 receptors (MC4) is a key common mechanism mediating the increases in renal sympathetic nerve activity (RSNA) induced by leptin and insulin (Rahmouni et al., 2003), suggesting the potential for an interaction on RSNA, such that increased levels of both hormones could result in an additive action on RSNA. Interestingly both insulin and leptin can regulate each other's production and release, such that an increase in plasma levels of insulin can lead to an increase in leptin levels and vice versa (Santos et al., 2000; Borer, 2014), which further suggests potential interactions of the two hormones. Thus, in order to explore the possibility that insulin and leptin interact within the brain to induce greater increases in RSNA, the first aim of the present study was to compare RSNA in response to administration of leptin and insulin alone and in combination.

The second aim was to explore potential sites where leptin and insulin may interact. Thus, we also quantified the number of activated neurons in brain regions, known to influence the sympathetic nervous system, and compared their distribution after central administration of leptin and insulin alone and both combined.

## Materials and methods

### Animals

Experiments were performed to comply with the Prevention of Cruelty to Animals Act 1986 (Australia). All experimental protocols conform with the “Guiding Principles for Research Involving Animals and Human Beings” and the guidelines set out by the “Australian Code of Practice for the Care and Use of Animals for Scientific Purposes,” 2013 (National Health and Medical Research Council of Australia) and were approved by the Royal Melbourne Institute of Technology (RMIT) University Animal Ethics Committee. Male Sprague–Dawley rats were obtained from the ARC Animal Resources Center (Western Australia, Australia). Rats were housed under a 12:12 h light/dark cycle at 23°C and allowed free access to standard rat chow and water.

### Procedures

#### General

General anesthesia was induced using isoflurane in O_2_ (2.5–5%) on the day of the experiment to allow catheterization of the femoral vein and artery. The catheter was inserted into the femoral vein, so that general anesthesia could be maintained with intravenous urethane (1.4–1.6 g/kg initially, followed by supplemental doses of 0.05 ml of a 25% solution if required). The depth of anesthesia was maintained to ensure the absence of corneal and pedal pinch reflexes. The femoral artery was cannulated for arterial blood pressure monitoring. Mean arterial pressure (MAP) and heart rate (HR) were calculated from the arterial pressure pulse using LABCHART (ADInstruments, Castle Hill, NSW, Australia). Rats were kept on a heating pad for the duration of the experiments to maintain body temperature at ~37.5°C.

#### Renal sympathetic nerve activity

A flank incision was made to expose the left kidney retroperitoneally. Using an operating microscope, the renal nerve was carefully dissected free of surrounding tissue. Under mineral oil, the renal nerve was then cut and the proximal end was placed onto the bared tips of two Teflon-coated silver wire electrodes.

The RSNA was amplified using a low-noise differential amplifier (models ENG 187B and 133, Baker Institute, Victoria, Australia), filtered (band pass 100–1000 Hz), rectified, and integrated at 0.5 s intervals. The signal was recorded using a PowerLab data acquisition system (ADInstruments, NSW, Australia).

#### Microinjections into the lateral brain ventricle (ICV)

Animals were placed prone, and their heads were mounted in a Stoelting stereotaxic frame, so that bregma and lambda were positioned on the same horizontal plane. To expose the dorsal surface of the brain, a hole (diameter 2 mm) centered 0.7 mm caudal and 1.4 mm lateral from bregma, was drilled into the skull. After the drilling procedure, the hole was covered with cotton wool soaked in normal saline to prevent drying of the exposed surface. A microinjection was made unilaterally using a fine glass micropipette (tip diameter 50–70 μm) inserted into the lateral brain ventricle (stereotaxic coordinates: 0.7 mm caudal to bregma, 1.4 mm lateral to midline, and 3.7–3.9 mm ventral to the surface of the dura). After the microinjection, the micropipette was left in place for 1 min. At the end of the experiment, a small amount of pontamine sky blue was microinjected using the same coordinates to confirm microinjection into the lateral ventricle.

#### Experimental protocols

MAP, HR and RSNA were measured prior to intracebroventricular (ICV) injection of vehicle (saline 5 μl; *n* = 5), leptin (7 μg in 5 μl; *n* = 5) or insulin (500 mU in 5 μl; *n* = 4) alone or in combination [leptin (7 μg in 5 μl) was administered 15 min after insulin (500 mU in 5 μl) (*n* = 4)], and the responses were monitored for 3 h after the last injection. Apart from instances in which leptin and insulin were administered in the one animal, only one injection per rat was performed. The doses of each hormone were chosen on the basis of previous studies (Rahmouni et al., 2009; Kosari et al., 2011). The doses used are larger than those observed in plasma as noted previously (Azuma et al., 2003; Degawa-Yamauchi et al., 2003; Tovar et al., 2005; Kosari et al., 2012). The difference in amounts is likely to be related to the concentration required at the actual site of action.

#### Immunohistochemistry

At the end of the experiment, the animals were perfused with 200 ml of 0.1 M phosphate buffered saline (PBS, pH 7.2) transcardially, followed with 200 ml of 4% *para*-formaldehyde in 0.1 M PBS. The brains were removed carefully and post-fixed for 3–3.5 h and then transferred to a solution of 20% sucrose in PBS 0.1 M and stored at 4°C.

Brains were sliced into a series of coronal sections (40 μm thick) using a cryostat (Leica, CM1900). Sections were collected and left in cryoprotectant until processed. One in five sections were then collected and processed immunohistochemically as free floating sections for the detection of Fos protein. In brief, the process involved placing the sections in 0.5% H_2_O_2_ for 15 min to destroy endogenous peroxidase activity. This was followed by incubation in 10% normal horse serum (NHS) and 0.5% Triton X-100 for 60 min to facilitate antibody penetration. Sections were then incubated in the primary antibody raised in rabbit against Fos (1:5000, rabbit polyclonal IgG, c-Fos (K-25): sc-253, Santa Cruz Biotechnology, CA, USA;) overnight at 4°C. Sections were then incubated with biotinylated anti rabbit secondary antibody raised in horse (1:400, Sigma Aldrich, Australia) for 90 min. This was followed by Extravidin–peroxidase complex (1:400, Sigma Aldrich, Australia) for 45 min. Sections were washed three times in 0.1 M PBS (5 min each time) between each incubation. Sections were then placed for 10 min in 0.01% 3,3′-diaminobenzidine hydrochloride (DAB; sigma Aldrich, Australia) + 0.02% Nickel ammonium + 0.02% Cobalt chloride in PBS. 5 μl of 30% hydrogen peroxide was added to begin the reaction. To end the reaction the sections were washed with PBS and then they were placed onto gelatine-coated slides and left overnight to dry, followed by coverslipping with Depex (Sigma Aldrich Australia) the next day.

### Statistical analysis

#### Physiological parameters

The resting levels of MAP and HR, and RSNA before each injection were averaged from three 5 min recordings taken 15–30 min prior to the first injection. The average levels were compared between groups using one-way ANOVA. The integrated RSNA, averaged over 1–2 min, was calculated at 15 min intervals over the period of 180 min, and expressed as a percentage of the resting level before the drug injections. Changes in MAP, heart rate, and RSNA were compared between groups in each experimental series by using two-way ANOVA. All results are expressed as the mean ± *SE*. *P* < 0.05 was considered statistically significant.

#### Immunohistochemistry

Under a bright field illumination, Fos-positive cell nuclei were visualized and counted unilaterally, except in midline structures [organum vasculosum of the lamina terminalis (OVLT), medium preoptic nucleus and raphe pallidus] at 200 × magnification. At least two sections per brain nucleus were examined in each animal. The average number of Fos-positive cell nuclei per section was calculated for each brain region in each animal and comparisons between the groups were performed using one-way ANOVA.

For both the physiological and immunohistochemical data, following the detection of an overall significant difference between groups, comparisons between groups administered the hormones and control, and between leptin + insulin combined vs. leptin or insulin alone, were made using Holm-Sidak's multiple comparison test. All results are expressed as means ± SE. *P* < 0.05 was considered to be statistically significant.

PRISM software was used.

## Results

### Effects of ICV injection of leptin and insulin on RSNA

**Figure 1:**
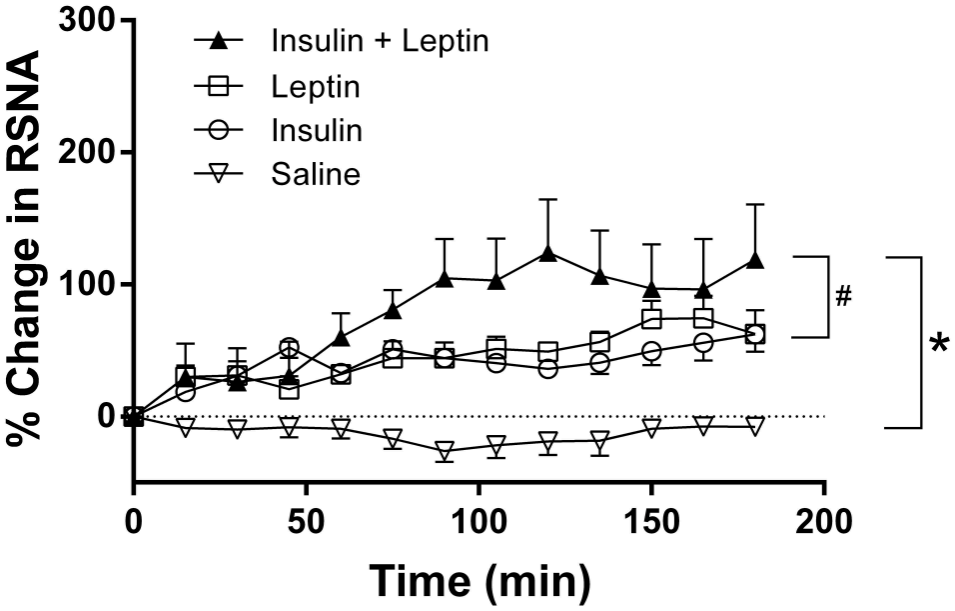
**Percent changes in renal sympathetic nerve activity (RSNA) from baseline levels over time after intracerebroventricular injection of leptin (*n* = 5) (7 μg/5 μl), insulin (*n* = 4) (500 mU/5 μl), saline (*n* = 5) (5 μl) and the combination of leptin and insulin (*n* = 4)**. ^*^P < 0.0001 leptin alone, insulin alone and both combined compared to saline; ^#^*P* < 0.001 insulin alone compared to leptin and insulin combined or leptin alone.

Following ICV injection of leptin alone RSNA increased significantly by 74 ± 17% (*P* < 0.0001 compared to saline; Figure 1). When insulin was administered alone the increase in RSNA was similar to that of leptin reaching a maximum of 62 ± 13% (*P* < 0.0001 compared to saline; Figure 1). Following the ICV administration of the combination of leptin and insulin the increase in RSNA was much greater reaching a maximum response of 124 ± 40% by 120 min (*P* < 0.0001 compared to control). The marked increase in RSNA was significantly greater than that observed after each hormone was administered alone (*P* < 0.0005; Figure 1).

### Effects of ICV injection of leptin and insulin on map and HR

**Figure 2:**
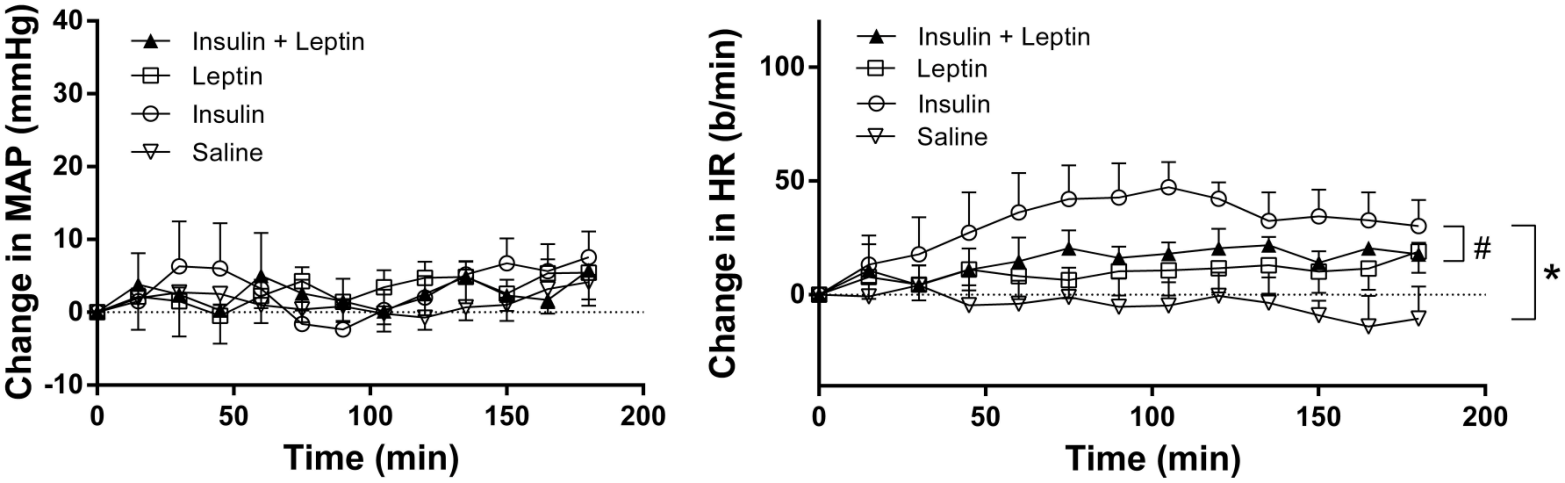
**Changes in mean arterial pressure (MAP) and heart rate (HR) over time induced by the intracerebroventricular administration of leptin (*n* = 5) (7 μg/5 μl), insulin (*n* = 4) (500 mU/5 μl), saline (*n* = 5) (5 μl) and the combination of leptin and insulin (*n* = 4)**. ^*^P < 0.0001 Leptin alone, insulin alone and both combined compared to saline; ^#^*P* < 0.0005 leptin and insulin combined compared to insulin alone.

Compared to saline, there were no marked changes in MAP during the 3 h observation period following leptin and insulin administered alone or combined (Figure 2). However, HR increased significantly following insulin alone compared to saline (*P* < 0.0001) (Figure 2). Leptin alone also significantly increased HR compared to saline (Figure 2). When insulin and leptin were combined, the HR response was significantly greater than saline (*P* < 0.0001), but significantly less than that of insulin alone (*P* < 0.001) (Figure 2). The response was similar to that of leptin alone. Thus, the presence of leptin attenuated insulin's tachycardic actions (Figure 2).

Resting MAP and HR prior to the ICV injections are shown in Table 1. No significant differences were detected between the groups (Table 1).

**Table 1:** **Resting mean arterial pressure (MAP) and heart rate (HR) prior to intracerebroventricular injection of saline (*n* = 5), leptin (7 μg, *n* = 5), insulin (500 mU, *n* = 4) and the combination of leptin and insulin (*n* = 4) in anesthetised rats**.

	**Saline**	**Leptin**	**Insulin**	**Leptin + Insulin**
MAP (mmHg)	93.5 ± 4.1	79.7 ± 1.6	88.0 ± 6.8	86.2 ± 5.6
HR (beats/min)	360 ± 7	336 ± 11	312 ± 19	318 ± 28

### Effects of leptin and insulin on Fos-positive cell nuclei

#### Lamina terminalis

**Figure 3:**
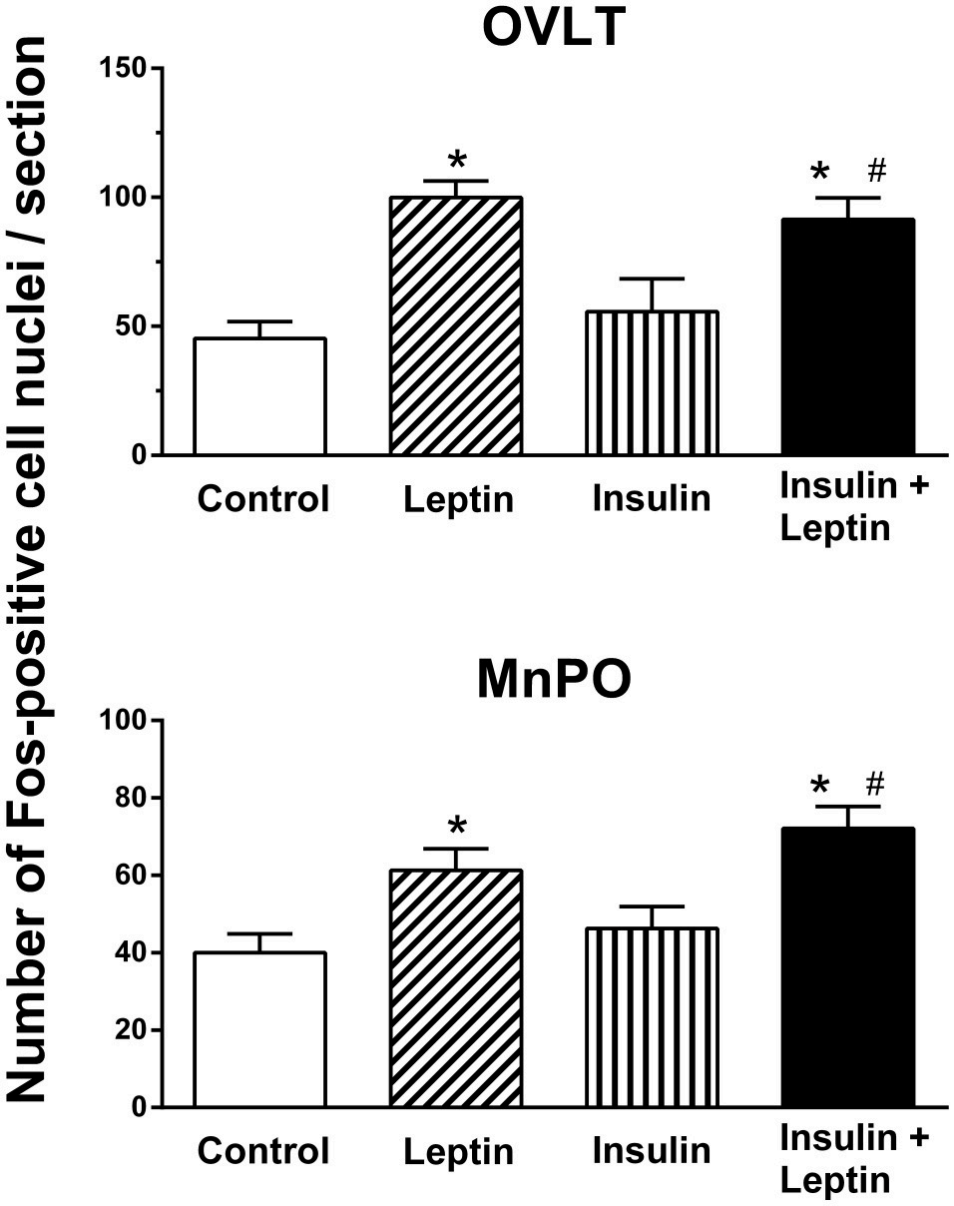
**Average number of Fos-positive cell nuclei per section in the organum vasculosum of lamina terminalis (OVLT) and median preoptic nucleus (MnPO) from rats administered intracerebroventricular saline (control) (*n* = 5) (5 μl), leptin (*n* = 5) (7 μg/5 μl), insulin (*n* = 4) (500 mU/5 μl), and the combination of leptin and insulin (*n* = 4)**. ^*^P < 0.01 compared to saline; ^#^*P* < 0.05 compared to insulin alone.

The numbers of Fos-positive cell nuclei in the OVLT following ICV injection of insulin alone were not significantly different compared to saline (control) (Figure 3). However, in this brain region, leptin alone significantly increased the number of Fos-positive cell nuclei compared to control (100 ± 6 vs. 45 ± 7/section, *P* < 0.01) (Figure 3). When leptin and insulin were combined the number of Fos-positive cell nuclei observed almost doubled (91 ± 8) compared to control and was significantly greater than that observed following insulin alone (*P* < 0.05) (Figure 3). The response following combined leptin and insulin was similar in magnitude to that following leptin alone (Figure 3). Thus, insulin did not influence the response to leptin in the OVLT.

In the median preoptic (MnPO) nucleus, similar effects were observed; insulin alone did not significantly affect the number of Fos-positive cell nuclei compared to the saline control (Figure 3) but there was a significant increase in Fos production after ICV leptin alone (Figure 3). The administration of leptin and insulin in combination also significantly increased the number of Fos-positive cell nuclei compared to control (*P* < 0.01) (Figure 3). This increase in Fos production was similar to that observed after leptin alone (Figure 3). However, the response to the combination of insulin and leptin was significantly greater that that observed following insulin alone (*P* < 0.05) (Figure 3).

#### Hypothalamus

##### Arcuate nucleus

**Figure 4:**
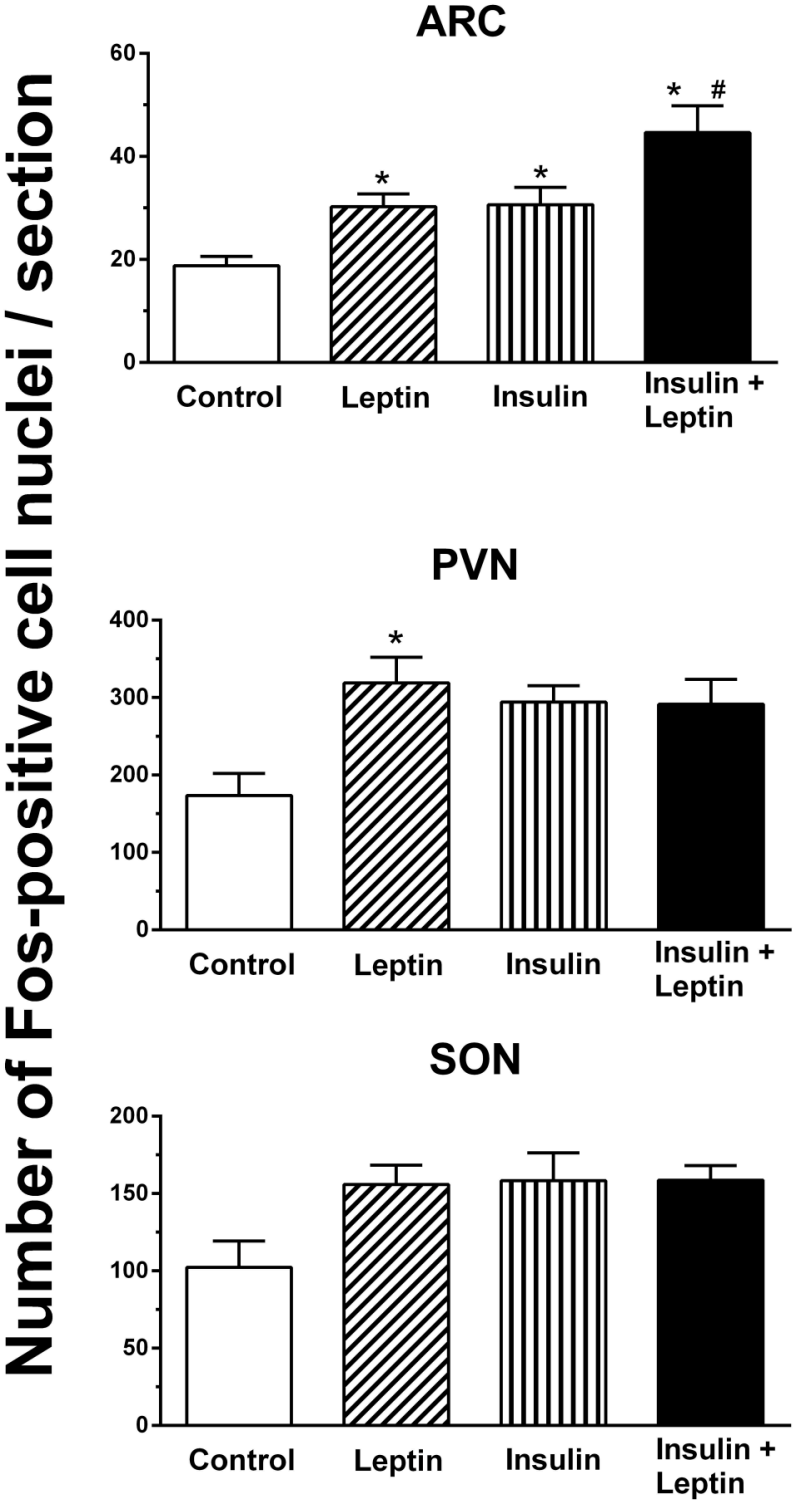
**The average number of Fos-positive cell nuclei per section in the arcuate (ARC), paraventricular (PVN) and supraoptic (SON) nuclei after intracerebroventricular injection saline (control) (*n* = 5) (5 μl), leptin (*n* = 5) (7 μg/5 μl), insulin (*n* = 4) (500 mU/5 μl), and the combination of leptin and insulin (*n* = 4)**. ^*^P < 0.01 compared to saline; ^#^*P* < 0.05 compared to insulin alone.

**Figure 5:**
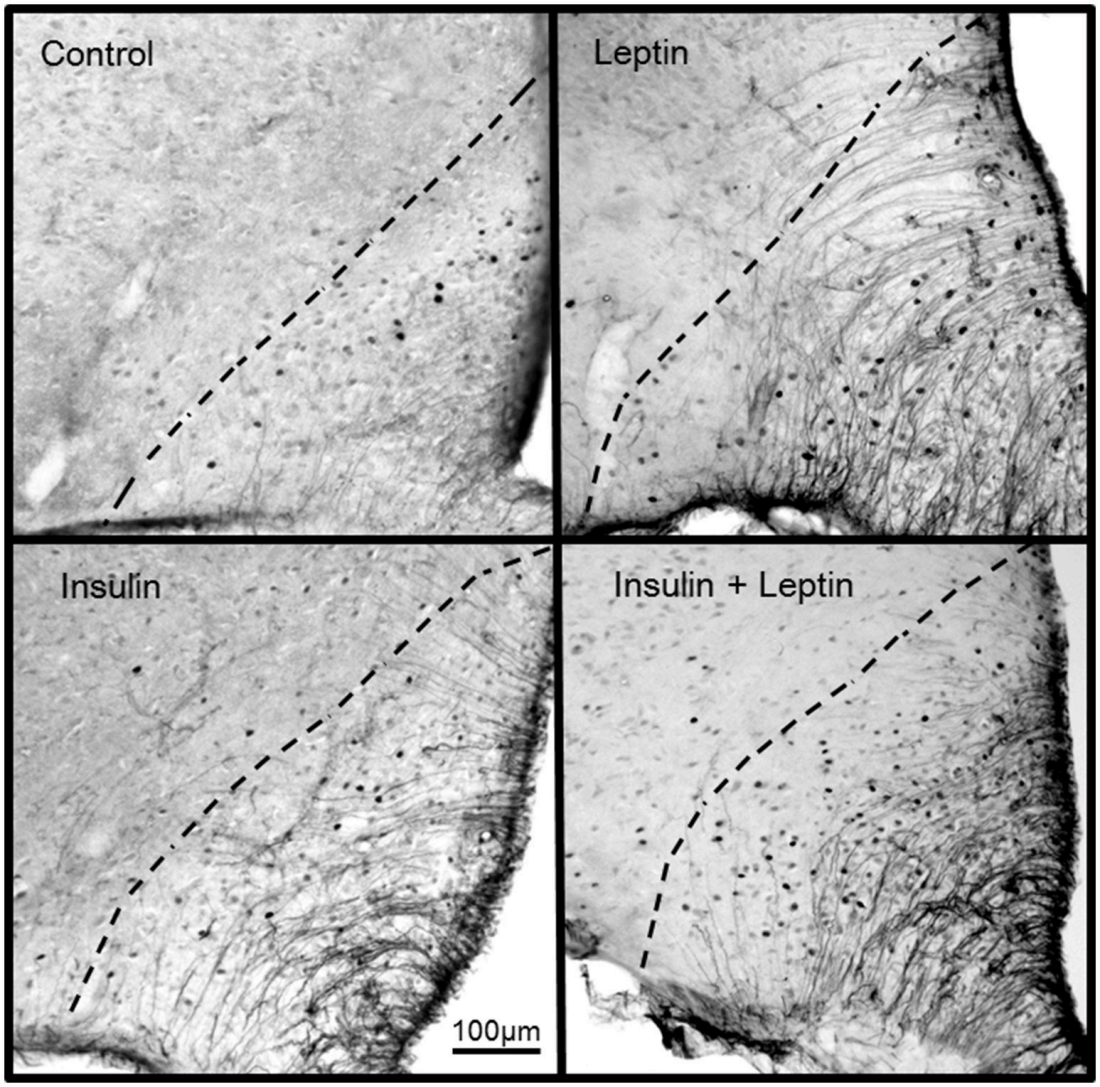
**Photomicrographs showing Fos-positive cell nuclei in the arcuate nucleus of rats following centrally administered saline (5 μl; control), leptin (7 μg), insulin (500 mU) and leptin combined with insulin**. Bar = 100 μm. Dashed lines outline the arcuate nucleus. The third ventricle is to the right of each photomicrograph.

In the arcuate nucleus (ARC), leptin alone and insulin alone significantly increased the number of Fos-positive cell nuclei compared to control (*P* < 0.05) (Figure 4). The combined administration of leptin and insulin produced a significantly greater increase in the number of Fos-positive cell nuclei in the ARC compared with either hormone alone (*P* < 0.05) (Figures 4, 5). Thus, the response to the combined administration of insulin and leptin was enhanced compared to the responses of the individual hormones.

##### Paraventricular and supraoptic nuclei

In the hypothalamic paraventricular nucleus ICV administration of leptin alone induced a significant increase in the number of Fos-positive cell nuclei compared to control (Figure 4). However, insulin alone or the combination of leptin and insulin did not attain statistically significant increases compared to control (Figure 4).

In the supraoptic nucleus, although a significant difference between the treatment groups was detected overall (*P* < 0.05), the analysis of the individual comparisons between a treated group (i.e., leptin, insulin alone, or combined) compared to control did not identify a statistically significant difference (Figure 4).

#### Medulla oblongata

**Figure 6:**
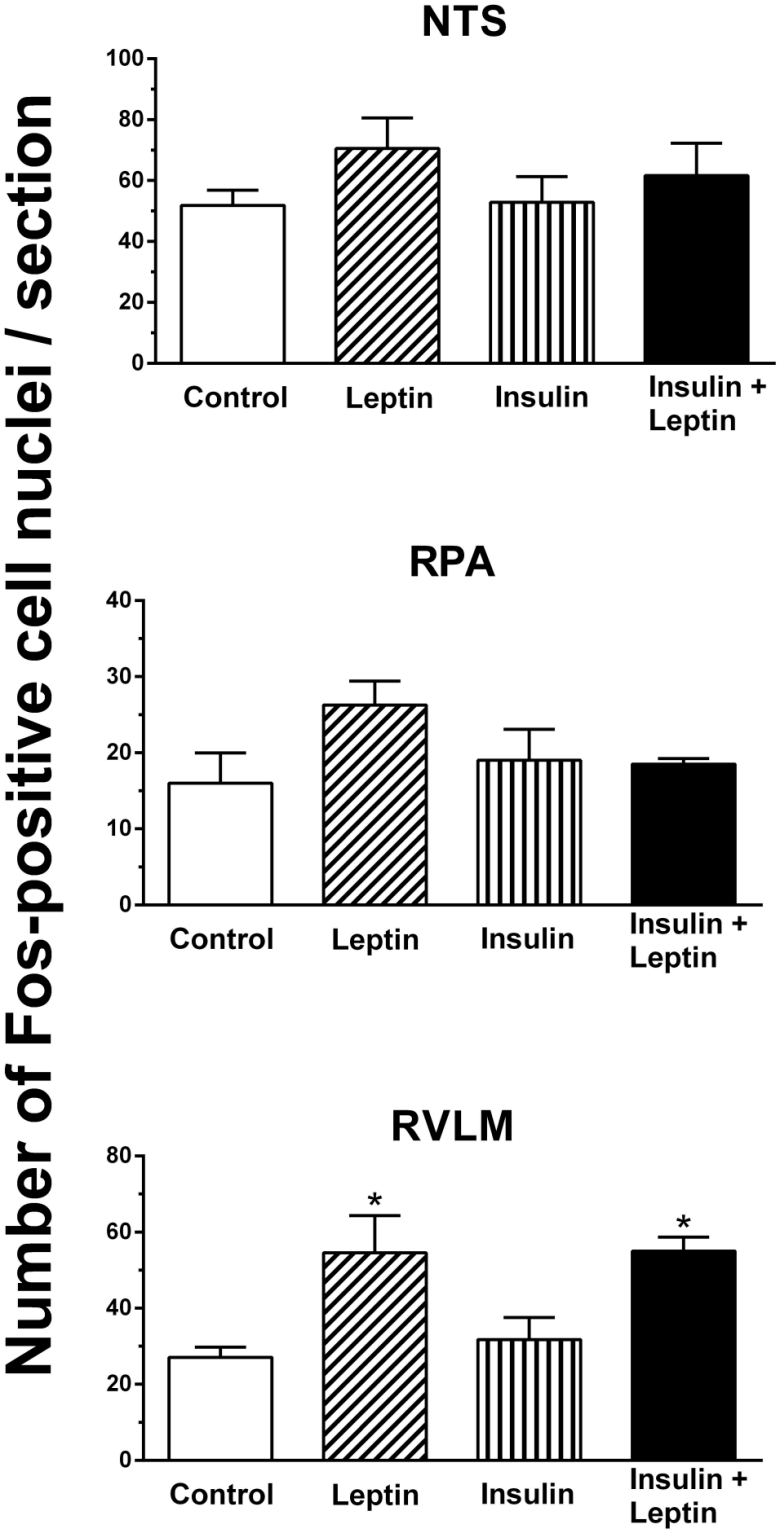
**Average number of Fos-positive cell nuclei per section in the nucleus tractus solitarius (NTS), raphe pallidus (RPA) and rostral ventrolateral medulla (RVLM), following saline (control) (*n* = 5) (5 μl), leptin (*n* = 5) (7 μg/5 μl), insulin (*n* = 4) (500 mU/5 μl), and the combination of leptin and insulin (*n* = 4)**. (^*^P < 0.05 compared to saline).

Fos-positive cell nuclei were examined in the nucleus tractus solitarius (NTS), raphe pallidus (RPA), and rostral ventrolateral medulla (RVLM) in the medulla oblongata. In the NTS and in the RPA there were no significant differences between groups overall. However, in the RVLM there was a significant difference between the groups (*P* < 0.05). There was a significant increase in the number of Fos-positive cell nuclei following the administration of leptin alone (*P* < 0.05) but not with insulin alone (Figure 6). Following the combination of leptin and insulin the increase in Fos-positive cell nuclei was similar to that of leptin and two-fold greater than in control (*P* < 0.05 compared to control) (Figure 6). Thus, insulin did not affect the increase induced by leptin in the RVLM.

## Discussion

This report is the first to demonstrate that the ICV administration of leptin combined with insulin results in significantly greater increases in RSNA compared with either hormone administered alone. The sympatho-excitatory effect did not appear to be a generalized response since the combined administration of leptin and insulin did not result in an increased HR response, rather the tachycardic effects elicited by insulin alone were attenuated by the presence of leptin. We also investigated brain nuclei involved in autonomic regulation to determine whether there was a correlation with those responses and increased neuronal activation (detected by the presence of Fos-positive cell nuclei) when leptin and insulin were administered. In the regions we investigated, we found that in the ARC, OVLT, MnPO, and RVLM the number of Fos-positive cell nuclei were significantly greater following ICV injection of leptin and insulin together compared to control. However, only in the ARC was the increase significantly greater than insulin or leptin administered alone.

The present work is the first to show a greater sympatho-excitatory response when leptin and insulin are present together compared to the responses elicited by each alone. The present work confirms previous studies that have shown significant increases in RSNA following the central administration of leptin alone or insulin alone (Dunbar et al., 1997; Rahmouni and Haynes, 2004; Morgan et al., 2008; Morgan and Rahmouni, 2010). The significance of our current work is that it highlights the potential for a much greater sympatho-excitatory effect on RSNA in conditions in which leptin and insulin are elevated, such as metabolic syndrome and obesity (Haynes et al., 1997 p. 2983; Paracchini et al., 2005; Schwartz and Porte, 2005; Prior et al., 2010). In those conditions, RSNA is abnormally elevated and leptin is believed to make a critical contribution to the increase (Simonds et al., 2014). Our findings suggest that the considerable contribution of leptin to this elevated RSNA may be, in part, due to the presence of increased levels of insulin.

In the present study we found that central administration of insulin increased heart rate, however, the response was prevented when leptin was present. Since the changes in heart rate elicited by central insulin are mediated by increases in sympathetic nerve activity (Cabou et al., 2007), the inhibition of this effect when leptin was present contrasts dramatically with the observed effect on RSNA, and suggests that the central interactions between leptin and insulin on the sympathetic nervous system are complex and may reflect differences in the output to different organs such that effects on the renal output is an additive response whilst that to the heart is an antagonistic interaction.

There was no effect on MAP following the ICV administration of leptin or insulin administered alone or in combination, suggesting that increases in RSNA are not sufficient to acutely increase MAP. The present findings are consistent with previous reports that found that acute ICV administration of leptin did not increase MAP over the time frame of our observations (Dunbar et al., 1997; Rahmouni and Morgan, 2007). Similarly, central administration of insulin has also been reported to have little effect acutely on blood pressure (Baron et al., 1993; Vollenweider et al., 1993; Rahmouni and Haynes, 2004).

In contrast, effects on MAP following chronic systemic or central administration, for 3–10 days, of leptin or insulin alone have been more consistent reporting significantly increased MAP responses (Meehan et al., 1994; Shek et al., 1998; Dubinion et al., 2011). Since renal function is an important determinant in the regulation of chronic blood pressure, the possibility exists that if the effect of the acute combination of leptin and insulin on RSNA could be reproduced with chronic infusions, then increases in MAP in conditions in which both hormones are chronically elevated may occur, but this awaits further investigation.

Previous work on interactions between leptin and insulin has focussed on metabolic functions, with little investigation into interactions on cardiovascular function. In particular, there is a distinct gap investigating an interaction on the cardiovascular responses arising from leptin and insulin acting within the brain, despite the fact that the central mechanisms that mediate the increase in RSNA induced by leptin or insulin alone have a common pathway that involves the activation of MC4 receptors (Rahmouni et al., 2003). This strongly suggests that an interaction is likely, and the present study now provides evidence to support a central interaction on RSNA.

The central interaction of insulin and leptin may not be restricted to the renal output. Evidence indicates that increases in lumbar sympathetic nerve activity induced by centrally administered insulin involves PI3K as is the case with leptin, whilst the increase in sympathetic nerve activity to brown adipose tissue induced by leptin or insulin involves activation of ERK1/2 (Rahmouni and Haynes, 2004). Thus, an interaction between leptin and insulin influencing at least three sympathetic outputs is a distinct possibility.

In the present study we investigated the distribution of activated neurons using the protein marker, Fos, in cell nuclei in brain regions known for their contribution to cardiovascular regulation. Only in the ARC was the number of activated neurons significantly greater following the combination of leptin and insulin than the increase seen after either hormone alone. This result is in agreement with reports that show leptin and insulin act within the ARC to increase sympathetic nerve activity (Elias et al., 1999; Cowley et al., 2001; Rahmouni et al., 2003; Rahmouni and Haynes, 2004; Rahmouni and Morgan, 2007; Morgan and Rahmouni, 2010; Cassaglia et al., 2011; Harlan et al., 2011; Ha et al., 2013). Thus, the activation of neurons in the ARC would be in accord with the increase in RSNA induced by leptin or insulin. The greater number of activated neurons in the ARC after the administration of leptin and insulin in combination correlates with the greater increase in RSNA compared to each hormone alone that we observed.

We found a significant increase in Fos-positive cell nuclei in the lamina terminalis (OVLT, MnPO), hypothalamic PVN and in the RVLM following leptin administration. We did not detect a statistically significant increase following insulin in those same regions. Although, the PVN and RVLM are believed to be involved in mediating the sympatho-excitatory effects of hyperinsulinemia, microinjections of insulin directly into those brain nuclei did not induce an increased sympathetic nerve activity suggesting there is no direct activation of insulin receptors in those brain nuclei (Bardgett et al., 2010; Ward et al., 2011; Stocker and Gordon, 2015). Since we found that insulin alone increased RSNA, it was perhaps, surprising that we did not detect an increase in Fos in those regions. Whether the type of anesthesia used (e.g., urethane in this study and alpha chloralose in those mentioned above) contributes to this anomaly is unknown. In contrast, no anomaly appears to exist with leptin since it significantly increased Fos production in the PVN and RVLM. Other factors that could contribute to the greater basal Fos may include duration of the stimulus and the surgical intervention that has occurred. Additionally, it should be noted that subtle influences or changes in the nuclei that affect sympathetic outflow may not be easily visualized by the use of the immunohistochemical detection of Fos.

In the OVLT, MnPO, and RVLM the combined administration of leptin and insulin significantly increased Fos-positive cell nuclei but the effects were similar to leptin alone. Thus, in those brain nuclei, the results suggest that the increase in Fos-positive nuclei were due primarily to leptin and that that there was no evidence of an additive interaction between leptin and insulin in those brain nuclei. These findings contrast with the observations we made in the ARC, suggesting that interactions between leptin and insulin in the brain may occur in selective brain nuclei.

## Summary and conclusions

In summary, following ICV administration of leptin and insulin together there was a significantly greater increase in RSNA than either hormone alone. Insulin alone increased HR significantly but this was prevented by the presence of leptin. MAP was not significantly different between groups. Of the brain regions examined, only in the arcuate nucleus did the combination of leptin and insulin together increase the number of Fos-positive cell nuclei significantly more than the increases following leptin or insulin alone. In contrast, in the lamina terminalis and rostral ventrolateral medulla, leptin and insulin combined increased Fos-positive cell nuclei to levels similar to leptin alone. The results suggest that where leptin and insulin are elevated, there may be greater increases in RSNA. This may contribute to cardiovascular complications. The arcuate nucleus may be a common site of cardiovascular integration for leptin and insulin.

## Author contributions

The work was performed in the laboratory of EB. EB devised study, analyzed the data, interpreted the data and wrote manuscript. HH and NA, performed experiments, analyzed and interpreted the data and contributed to writing the manuscript. TJ and MS contributed to the manuscript and interpretation of the data. All authors approved the final version of the manuscript and agree to be accountable for all aspects of the work in ensuring that questions related to the accuracy or integrity of any part of the work are appropriately investigated and resolved. All persons designated as authors qualify for authorship, and all those who qualify for authorship are listed.

### Conflict of interest statement

The authors declare that the research was conducted in the absence of any commercial or financial relationships that could be construed as a potential conflict of interest.
